# Flow Cytometric DNA Analysis and Histopathologic Re-Evaluation of Paraffin Embedded Samples from Hydatidiform Moles and Hydropic Abortions

**DOI:** 10.22074/ijfs.2015.4547

**Published:** 2015-10-31

**Authors:** Narges Izadi-Mood, Soheila Sarmadi, Reza Tayebivaljozi, Farzaneh Mohammadi-Zia, Mohammad Farhadi

**Affiliations:** 1 Department of Pathology, Women Hospital, Tehran University of Medical Sciences, Tehran, Iran; 2 Department of Pathology, Baharlou Hospital, Tehran University of Medical Sciences, Tehran, Iran; 3 Department of Pathology, Iranian Blood Transfusion Organization, Shahid Hemmat Highway, Tehran, Iran

**Keywords:** Partial Hydatidiform Mole, Complete Hydatidiform Mole, Hydropic Abor- tion, Flow Cytometry

## Abstract

**Background:**

Distinction of hydatidiform moles (HMs) from non-molar abortions and sub-classification of HMs are important for clinical practice; yet, diagnosis based solely on morphology is affected by interobserver variability. The objective of this study was to determine the role of DNA flow cytometry in distinguishing molar from non-molar pregnancies.

**Materials and Methods:**

This retrospective study was conducted at the Department of Pathology, Women’s Hospital, Tehran University of Medical Sciences, Tehran, Iran, between 2006 and 2010. DNA ploidy analysis and histopathologic re-evaluation were performed on paraffin-embedded tissue from 36 (17 complete and 19 partial) molar and 24 hydropic abortus (HA) cases which were previously diagnosed based on histomorphologic study.

**Results:**

Of the 17 cases initially diagnosed as complete HM (CHM), 9 were diploid, 2 were triploid, 5 were tetraploid and 1 was aneuploid. Of the 19 initial partial HMs (PHMs), 2, 8, 1 and 8 cases were diploid, triploid, tetraploid and aneuploid, respectively. In the initial HA category (n=24), 14 diploid, 1 triploid, 5 tetraploid, and 4 aneuploid cases existed. Following flow cytometry and histopathologic reevaluation, 1 case with previous diagnosis of HA was reclassified as PHM, 2 initial PHMs were reclassified as CHM and 2 initial CHMs were categorized as PHM.

**Conclusion:**

The results show that correct diagnosis of PMH is the main challenge in histological diagnosis of gestational trophoblastic disease (GTD). DNA flow cytometric analysis could be an informative supplement to the histological interpretation of molar and hydropic placentas.

## Introduction

Hydatidiform mole (HM) is a complication of gestation observed in approximately 0.5-1/1000 pregnancies in the western world and most prevalent in South-East Asia with rates ranging from 1-2/1000 pregnancies in Japan and China to 12/1000 pregnancies in Indonesia, India and Turkey (1,3). HM is classified as either complete or partial based on morphological, histopathological, and cytogenetic studies (4). Genetically, complete HM (CHM) has diploid 46XX karyotype. Although chromosomes are entirely of paternal origin, mitochondrial DNA is of maternal origin. By contrast, partial HM (PHM) has diandric monogynic triploid karyotype (69 chromosomes), which is the "gold standard" for the ultimate diagnosis (4,6). Most reports on the pathological evaluation of early abortions have focused on differentiating between PHM and CHM and between PHM and non-molar hydropic abortus (HA). It is of importance to distinguish these entities because PHMs and CHMs known as risk factors for developing an aggressive clinical and biological behavior, whereas HAs don’t. Persistent gestational trophoblastic disease (GTD) is predicted to occur in 10-30% of CHMs, but only in 1-7% of PHMs (7,8) have been identified. 

In many cases, distinction between HM and HA can be made based on only morphological examination. However, absence of sufficient published standard morphological criteria, presence of atypical cases and early evacuation of molar pregnancy cause major difficulties in the histopathological diagnosis of HM with significant interobserver and intraobserver variability (9,10). To help differentiate between partial and CHMs, some ancillary techniques such as genotyping, DNA ploidy analysis and p57 immunohistochemistry have been developed and used. However, their application and interpretation are not without drawbacks. For example, p57 is the gene product of the paternally imprinted, maternally expressed gene, while immunostaining of p57 is helpful in confirming the diagnosis of CHM, but this useful marker cannot differentiate between PHM and other non-molar gestations (4,11). Several studies have recently applied this commercially available and cost-effective molecular genotyping method in cases of molar gestations to identify and compare the parental genetic contribution in the chorionic villi and in the maternal decidua. Flow cytometry is widely accepted as a rapid and easy test for ploidy evaluation with ability to analyze a large number (10,000-20,000) of random nuclei and with sufficient sensitivity to distinguish diploidy from triploidy, tetraploidy and non-tri/tetraploid aneuploidy (4,5,7,8,12,13). However, ploidy analysis cannot differentiate between non-molar digynic triploid gestations and PHM. Correlation between histology and ploidy improves diagnostic accuracy and concordance among pathologists. 

In the present study, previously diagnosed samples from molar and non-molar aborted pregnancies were histopathologically reviewed and analyzed by flow cytometry. The correlation between histological diagnosis and ploidy status was then evaluated. 

## Materials and Methods

We carried out a retrospective study of 60 selected specimens from all aborted conceptions at the Department of Pathology, Women’s Hospital, Tehran University of Medical Sciences, Tehran, Iran, between 2006 and 2010. Present study was a retrospective research, so we were not able to obtain a written informed consent from the participant. The research protocol was approved by the Ethics Committee of Tehran University of Medical Sciences, Tehran, Iran. Specimens were obtained from spontaneous abortions or from curettages carried out after detection of intrauterine death or HM by ultrasound examination. A 4-µm section of all specimens was stained using hematoxylin and eosin (H&E) stain. Number of slides ranged from 1 to 10 (average of 3 slides per case). All of the slides were reviewed regarding the histological criteria described by Paradinas et al. (14). Diagnosis of CHM was made by microscopic finding of enlarged edematous villi, prominent central cistern formation, and moderate to marked circumferential trophoblastic hyperplasia, often with cytologic atypia. Diagnosis of PHM was made based on presence of dual population of villi (large, irregular, and hydropic villi as well as small and fibrotic villi), cistern formation in some enlarged villi, markedly irregular villi with scalloped borders, trophoblastic hyperplasia and presence of inner cell mass (15). 

Flow cytometric DNA analysis was performed on formalin-fixed, paraffin-embedded tissue blocks. The selection criterion was the presence of both placental and maternal (decidual) tissue in such amount that DNA histograms could be obtained. For each case, a tissue block containing at least 90% chorionic villi and no more than 10% decidua and blood was selected. Maternal decidual tissue had to be present as the internal diploid control. Sixty blocks were analyzed. The technique of Hedley was used for DNA analysis. Briefly, 30 µm-sections were cut from each case and then deparaffinized with xylene and rehydrated. Sections were subsequently incubated at 37˚C for 45 minutes with 0.05% pepsin in normal saline to disaggregate the tissue and yield the nuclei. The cell suspension was filtered through a 50-µm steel mesh and centrifuged at 1500 rpm for 5 minutes. The pellet was washed and resuspended in propidium iodide solution (Sigma, USA). After 30 minutes of incubation at room temperature, the processed tissue was analyzed by flow cytometry (Coulter Electronics, Hialeah, FL, UK). Fluorescence intensity measured on a linear axis was regarded as proportional to the DNA content of individual cells, 10,000 of which were analyzed to produce each histogram. 

Cellular DNA content was determined with an Epics C flow cytometer (Coulter Electronics, Hialeah, FL, UK). Histograms of 10,000 cells were recorded and analyzed. Placenta was classified as tetraploid if the peak in the G2/M region represented greater than 25% of the cells and the DNA index (DI) was between 1.90 and 2.10. Furthermore with DI between 1.40 and 1.60, placenta was considered to be triploid. In this study, we use the simple qualifier "aneuploid" to define cases with non-tri/tetraploid aneuploidy (7,16). The gathered data was analyzed by Statistical Package for Social Sciences version 18.0 (SPSS, SPSS Inc., Chicago, IL, USA) software. 

## Results

Patients’ age ranged from 15 to 50 years (mean: 29.6 ± 2.3), while gestational age ranged from 6 to 24 weeks (mean: 9.5 ± 2.1). Based on histopathological review of the sections, diagnoses were made as CHM in 17, PHM in 19 and HA in 24 cases. DNA ploidy analysis in the 60 cases showed 25 diploid, 11 triploid, 11 tetraploid and 13 aneuploid (non tri/tetraploid) cases. Of the 17 cases histologically diagnosed as CHM, 9 were diploid, 2 triploid, 5 tetraploid and 1 aneuploid according to the yielded histograms. Two diploid, 8 triploid, 1 tetraploid and 8 aneuploid histograms were produced by the 19 histologically-diagnosed PHM cases. At last, of the 24 cases with histological diagnosis as HA, 14, 1, 5 and 4 cases yielded diploid, triploid, tetraploid and aneuploid histograms, respectively. After the release of flow cytometric results, all of the slides from cases in which the primary histological diagnosis did not match the flow cytometry analysis result in terms of expected ploidy were reviewed. Finally, according to both flow cytometric analysis results and histological criteria, out of total 60 cases, 17 cases were diagnosed as CHM (15 cases from the initial histopathology-based diagnostic category of CHM and 2 cases from the initial diagnostic category of PHM). Twenty cases were finally diagnosed as PHM (17, 2, and 1 cases from the initial diagnostic categories of PHM, CHM and HA, respectively). Twentythree out of the 24 cases with preliminary diagnosis of HA were confirmed after incorporation of flow cytometric data into the diagnostic criteria. Comparing the initial histomorphologybased diagnoses with the results obtained from flow cytometric analysis, five cases were found to have discordant results. In these cases, the original H&E stained sections and clinical data were reviewed along with the ploidy status. Finally, the histological diagnosis was revised in all of the 5 cases. A summary of the results is presented in table 1.

**Table 1:** Summary of the results of study


Initial histo-morphological Diagnostic categories	Flow cytomery result		Definite diagnosis considering flow cytometry result and histopathological re-evaluation			
	Ploidy	No. of cases	CHM n=17	PMH n=20	HA n=23	No definite diagnosis

CHM, n=17	Diploid	9	9	-	-	-
	Triploid	2	-	2*	-	-
	Tetraploid	5	5	-	-	-
	Aneuploid	1	1	-	-	-
PHM, n=19	Diploid	2	2*	-	-	-
	Triploid	8	-	8	-	-
	Tetraploid	1	-	-	-	1
	Aneuploid	8	-	-	-	8
HA, n=24	Diploid	14	-	-	14	-
	Triploid	1	-	1**	-	-
	Tetraploid	5	-	-	5	-
	Aneuploid	4	-	-	4	-


CHM; Complete hydatidiform mole, PHM; Partial hydatidiform mole, HA; Hydropic abortion, *; The number of cases with discordant initial and definite results are underlined and **; Case of tubal pregnancy.

## Discussion

It is well known that CHM, PHM and HA represent three independent conditions in terms of etiology, pathology, genetic characteristics, morphology and clinical aspects. In most cases, the diagnosis is straightforward (17). Biological variability and scarcity of available tissue, however, will sometimes cause difficulties in clinical and morphological differentiation between different pathologies, mainly between CHM versus PHM or PHM versus HA (18). No single criterion is enough to make this distinction. Therefore, methods that evaluate the ploidy (such as karyotyping as well as flow and image cytometry) have been used to distinguish HM from HA (19). Flow cytometry permits evaluation of cellular DNA content and is employed to determine the ploidy status of different lesions. In this regard, a significant further advantage has been developed as a technique for extracting DNA from formalin fixed-tissue (20). When it comes to molar pregnancies, flow cytometry has confirmed that the majority of CHMs are diploid and that most PHMs are triploid. Thus, flow cytometric analysis of hydropic abortions can be used as an adjunct to histopathologic criteria to differentiate among PHM, CHM and HA. The current study, using flow cytometry as an ancillary techniques to improve the accuracy of histopathological diagnosis, further reveals the difficulty in making the correct diagnosis in hydropic abortions (especially in early gestation) based on histopathology. This difficulty in distinction among PHM, CHM and HA was demonstrated by 5 (8.3%) of the cases in our study, in which the primary diagnoses changed following the acquisition of flow cytometric results. In a study done by Fukunaga et al. (21), nuclear DNA content of 219 hydropic and 68 nonhydropic placentas (as a control) were analyzed by flow cytometry in paraffin-embedded tissue. Based on flow cytometry and review of the histology, 10 PHM diagnoses were reclassified as HA, 1 PHM diagnosis was revised as CHM, 4 HA diagnoses were changed to PHM and 1 CHM diagnosis was corrected as PHM. They had 16 discrepant diagnoses and their results showed that the main difficulty in histological diagnosis of hydropic abortions is due to the diagnosis of PHM. There is a considerable overlap in the histological features of PHM and HA as well as of PHM and CHM which result in discordant diagnoses. In another study of Fukunaga et al. (22), 76 cases of hydropic placentas were retrieved and analyzed by flow cytometry. Out of 23 specimens originally diagnosed as CHM, 21 diagnoses were confirmed and 2 were revised as PHM; out of 22 initially diagnosed PHMs, the primary diagnosis was confirmed in 20 cases and was changed to HA in two; and out of 31 firstly diagnosed HAs, 20 diagnoses were confirmed, while 9 and 1 diagnoses were revised as PHM and CHM, respectively. Also most of the cases with discordant diagnoses were definitively or initially diagnosed as PHM. They also concluded that PHM is a common condition that goes underdiagnosed because of the usual subtle histologic changes.

In our study, nine from the 17 cases initially diagnosed as CHM were diploid; therefore, the primary diagnosis was confirmed. Two cases out of 17 were triploid which ended in having the revised diagnosis as PHM based on re-evaluation of histological features. Five and one cases out of 17 were tetraploid and aneuploid, respectively. In the latter cases, we did not change the initial diagnosis of CHM. It is very important to interpret the ploidy result in conjunction with histomorphologic study (20). Although the great majority of CHMs and PHMs have been reported to be diploid and triploid, respectively, exceptional cases including nonmolar digynic triploid abortion, triploid androgenetic CHMs, and tetraploid CHMs and PHMs have been seen as well. Ploidy analysis is not helpful in evaluation of these exceptional cases of molar and non-molar gestation, so a correct diagnosis can be made through karyotype analysis by cytogenetic study and/or p57 immunostaining (23). 

In the present study, eight from the 19 cases with primary histological diagnosis as PHM were triploid; therefore, the primary diagnosis was confirmed. Two out of the 19 cases were diploid in which review of H&E slides and clinical data resulted in change of diagnosis to CHM. One and eight out of the 19 cases with primary diagnosis of PHM were tetraploid and aneuploid, respectively. 

In histomorphological review, these cases failed to show all the characteristic features of PHM; however, all showed the combination of dual villous population, round or oval trophoblastic pseudoinclusions, and cistern formation. As known from previous studies, several conditions including early CHM, trisomies, non-molar HAs, digynic triploid abortions, and placental mesenchymal dysplasia can mimic PHM at the morphologic level (6). In these cases, accurate diagnosis is not possible without genotyping. Therefore, we could not make any definitive diagnosis in the nine tetraploid and aneuploid cases which were primarily diagnosed as PHM. Fourteen out of the 24 cases with histological diagnosis as HA were diploid with the same diagnosis of HA on histological re-evaluation. One case out of the 24 was triploid which, after the histological review, was reclassified as PHM. In our study, similar to those mentioned above, the most discordant results were seen in the diagnosis of PHM. Moreover, our flow cytometry analysis in 9 cases with initial diagnosis of HA showed abnormal ploidy (5 tetraploid and 4 aneuploid) which could not be related to any form of molar gestation with the regard to the histopathologic criteria for HMs. This is supported by the fact that a great proportion of early abortions with hydropic changes are due to chromosome abnormalities. Since karyotyping was not performed in these cases, we were unable to verify or refute this possibility. 

In present study, one case with original diagnosis of HA with triploidy in flow cytometry and revised diagnosis as PHM was a tubal pregnancy. In addition, 4 other cases of tubal pregnancy existed in HA group, diagnosis of which was confirmed by flow cytometry. Regarding these findings, there might be some relationship between abnormality in DNA ploidy with occurrence of ectopic pregnancy and presence of hydropic changes in these abnormal pregnancies. 

In equivocal cases, ploidy analysis in association with histomorphologic study may be useful. Techniques including immunohistochemical analysis of p57 expression and molecular genotyping are also helpful in improving the diagnosis of hydatidiform moles, but have the limitation of not being able to establish maternal/paternal contributions of chromosome complements. For example, a dilpoid result by karyotyping or DNA flow cytometry analysis cannot distinguish a CHM (androgenetic diploidy) from a diploid NM (biparental diploidy), and a triploid result cannot distinguish a PHM (diandric triploidy) from a triploid NM (digynic triploidy). Similarly, p57 immunostain cannot distinguish a PHM from a diploid (biparental) or triploid (digynic) non-molar (due to the presence of a maternal chromosome complement, all share the same pattern of p57 expression) (24). 

In planning our study, we considered the high incidence of gestational trophoblastic disease in our country as well as the challenge encountered in their correct diagnosis, particularly in differentiating between PHM and non-molar hydropic changes, even based on immunohistochemistry as a routine procedure. Furthermore, molecular techniques could not be routinely implemented due to high cost. Thus, although flow cytometry is not a novel technique, we think that this study demonstrates its potential role as a cost effective as well as an efficient adjunct diagnostic tool to differentiate between the etiologies of molar/hydropic abortion, especially between PHM and non-molar hydropic abortion. 

## Conclusion

As a rapid and accurate means for determination of nuclear ploidy, DNA flow cytometric analysis can surely contribute to confirmation of histopathological diagnosis in most cases of molar pregnancy. This would provide valuable information, regarding the characteristics related to the persistent disease, which cannot always be obtained by macroscopic or microscopic inspection alone.
